# Epigenetic regulator Lid maintains germline stem cells through regulating JAK-STAT signaling pathway activity

**DOI:** 10.1242/bio.013961

**Published:** 2015-10-21

**Authors:** Lama Tarayrah, Yuping Li, Qiang Gan, Xin Chen

**Affiliations:** Department of Biology, 3400 North Charles Street, The Johns Hopkins University, Baltimore, MD 21218-2685, USA

**Keywords:** Germline stem cell, Niche, Epigenetics, Histone demethylase, *Drosophila*

## Abstract

Signaling pathways and epigenetic mechanisms have both been shown to play essential roles in regulating stem cell activity. While the role of either mechanism in this regulation is well established in multiple stem cell lineages, how the two mechanisms interact to regulate stem cell activity is not as well understood. Here we report that in the *Drosophila* testis, an H3K4me3-specific histone demethylase encoded by *little imaginal discs* (*lid*) maintains germline stem cell (GSC) mitotic index and prevents GSC premature differentiation. Lid is required in germ cells for proper expression of the Stat92E transcription factor, the downstream effector of the Janus kinase signal transducer and activator of transcription (JAK-STAT) signaling pathway. Our findings support a germ cell autonomous role for the JAK-STAT pathway in maintaining GSCs and place Lid as an upstream regulator of this pathway. Our study provides new insights into the biological functions of a histone demethylase *in vivo* and sheds light on the interaction between epigenetic mechanisms and signaling pathways in regulating stem cell activities.

## Abstract (precis)

**Summary:** This study provides new insights into the biological functions of a histone demethylase and sheds light on the interaction between epigenetic mechanisms and signaling pathways in regulating stem cell activities.

## INTRODUCTION

Extrinsic signals from cells comprising the stem cell niche are essential in maintaining stem cell activity (Morrison and Spradling, 2008). In addition, epigenetic regulation that changes stem cell chromatin structure without altering DNA sequences acts as an important intrinsic mechanism to maintain stem cells (Eun et al., 2010). Although both mechanisms are important to regulate stem cell activities, our understanding of the crosstalk between the two events is limited to a few examples (Cherry and Matunis, 2010; Eliazer et al., 2011; Tarayrah et al., 2013).

**Fig. 1.**
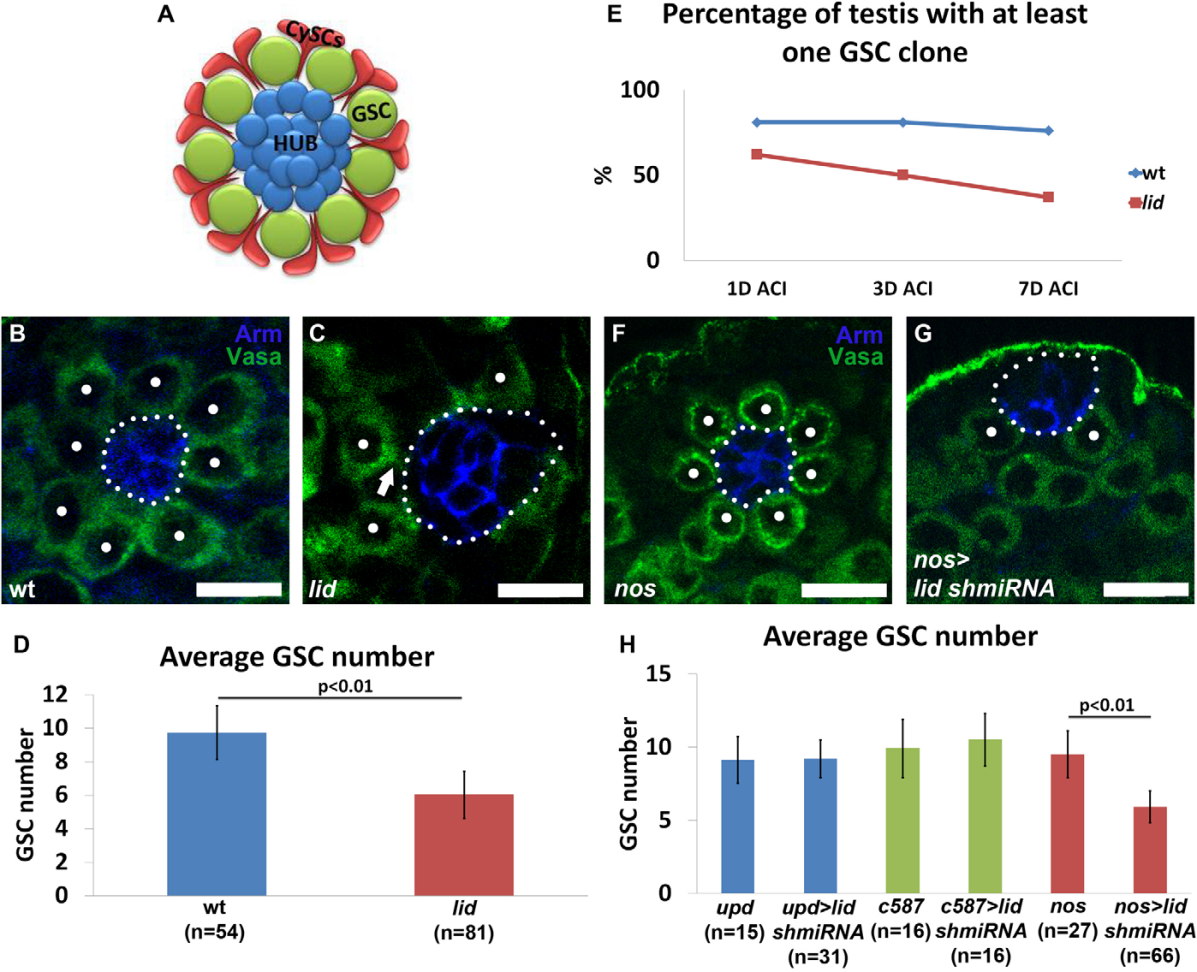
**Lid acts cell autonomously in the germline to maintain GSC number at the niche.** (A) Schematic of the *Drosophila* testis niche. CySCs, cyst stem cells; GSC, germline stem cell. (B,C,F,G) Immunostaining using antibodies against Armadillo (Arm) (blue) and Vasa (green) in wt (B), *lid* (C), *nos-Gal4* (F) and *nos-Gal4/UAS-lid shmiRNA* (G) testes. Arrow points to detached GSCs in *lid* (C) testes. Dots indicate GSCs which we identified as Vasa-labeled cells in direct contact with the hub. Hub area is outlined (white dotted line). (D) Quantification of average GSC number: 9.7±1.6 in wt testes vs 6.03±1.4 in *lid* testes. (E) Quantification of the percentage of testes with at least one GFP-negative *lid* GSC clone, one, three, and seven days after clone induction by heat shock in wt and *lid* testes. 1D ACI: wt (*n*=21), *lid* (*n*=37), *P*>0.05; 3D ACI: wt (*n*=21), *lid* (*n*=40), *P*<0.01; 7D ACI: wt (*n*=21), *lid* (*n*=38), *P*<0.01. *P*-value calculated by Fisher's test. (H) Quantification of average GSC number in testes from males of the following genotypes: *upd-Gal4* control (9.1±1.6); *upd-Gal4;; UAS-lid shmiRNA* (9.2±1.3); *c587-Gal4* control (9.9±2); *c587-Gal4;; UAS-lid shmiRNA* (10.5±1.8); *nos-Gal4* control (9.5±1.6); and *nos-Gal4/UAS-lid shmiRNA* (5.9±1.1). *P-*value calculated using Student's *t*-test. Error bars represent s.d. Scale bars: 10 µm.

*Drosophila* spermatogenesis is a paradigmatic system to investigate the molecular mechanisms responsible for the maintenance of adult stem cell activities in their physiological environment (Kiger et al., 2001; Tulina and Matunis, 2001; Yamashita et al., 2003, 2007). The *Drosophila* male germline stem cell (GSC) niche is one of the best characterized niches in which GSCs associate with two types of somatic cells: hub cells located at the tip of the testis, and cyst stem cells (CySCs) two of which surround each GSC (Fig. 1A). Hub cells and CySCs contribute to a niche that provides the critical signaling necessary to preserve GSC identity and activity (Kiger et al., 2001; Leatherman and DiNardo, 2008, 2010; Tulina and Matunis, 2001). Janus kinase signal transducer and activator of transcription (JAK-STAT) and bone morphogenetic protein (BMP) signaling pathways are the two major pathways involved in the maintenance of the male GSC niche. Activation of the JAK-STAT pathway is initiated by the secretion of the cytokine Unpaired (Upd) from the hub cells. Upd binds the receptor Domeless activating Hopscotch (Hop), the JAK kinase homolog in *Drosophila*, and Stat92E, the STAT homolog, in both GSCs and CySCs (reviewed in Hou et al., 2002). The intrinsic activation of Stat92E in CySCs activates BMP signaling and is thought to be sufficient to cause continuous GSC self-renewal while Stat92E in the GSCs has been reported to only regulate their adhesion to the niche (Leatherman and DiNardo, 2008, 2010).

In addition to signaling pathways, the chromatin structure of GSCs can profoundly influence critical decisions of stem cell maintenance versus differentiation. In *Drosophila*, histone modifying enzymes and chromatin remodeling factors are important chromatin regulators (Becker and Horz, 2002). Both have been shown to act cell autonomously to maintain GSCs in the testis niche (Buszczak et al., 2009; Cherry and Matunis, 2010; Eliazer et al., 2014). Among the histone modifying enzymes, histone demethylases have been identified as ‘epigenetic erasers’ that remove methyl-groups from methylated Lysine residues of histones (Klose et al., 2006). Specifically, the repressive trimethylation on lysine 27 of histone H3 (H3K27me3) and the active H3K4me3 histone modifications have been the focus of many studies. While H3K27me3 is laid down by a member of the Polycomb Group (PcG) proteins and associates with silent gene regions (Cao et al., 2002; Czermin et al., 2002; Kuzmichev et al., 2002; Muller et al., 2002), H3K4me3 is generated by the Trithorax Group (TrxG) family of proteins and has been shown to associate with active regions of chromatin (Byrd and Shearn, 2003). A recent study from our lab on the function of dUTX, a histone demethylase that targets the repressive H3K27me3 modification, revealed that dUTX regulates JAK-STAT pathway activity in the CySCs to maintain proper niche structure and gene expression (Tarayrah et al., 2013).

Among the 14 demethylases in *Drosophila* (Klose et al., 2006), *little imaginal discs* (*lid*) encodes a demethylase that specifically removes the active H3K4me3 modification (Eissenberg et al., 2007; Lee et al., 2007). Lid belongs to the JARID1 family of H3K4me3 demethylases (Secombe and Eisenman, 2007). While human cells encode four JARID1 family members (JARID1a, JARID1b, JARID1c, and JARID1d), Lid is the sole *Drosophila* homolog (Eissenberg et al., 2007). Previous studies have shown that *lid* mutant adult flies have increased global H3K4me3 levels (Eissenberg et al., 2007; Lee et al., 2007) and demonstrated a role for Lid in dMyc-induced cell growth (Li et al., 2010). However, the function of Lid in an endogenous stem cell system has not yet been elucidated. The mammalian Lid homologue JARID1a was reported to play a critical role in breast cancer metastatic progression, suggesting a role for H3K4me3 demethylases in inhibition of tumor progression and metastasis (Cao et al., 2014). Therefore, understanding the functions of Lid in an adult stem cell system might facilitate the targeting of histone demethylases for cancer treatment. Here we report that Lid is required cell autonomously to prevent premature differentiation of male GSCs by maintaining Stat92E levels. Our findings support a cell autonomous role for the JAK-STAT pathway in maintaining GSCs and provide insight into the *in vivo* functions of a histone demethylase.

## RESULTS

### Lid acts cell autonomously in the germline to maintain GSC number at the niche

The *lid* gene encodes a histone demethylase that has been reported to specifically demethylate H3K4me3 *in vivo* (Eissenberg et al., 2007; Lee et al., 2007). To confirm the function of Lid as a specific H3K4me3 demethylase in the testis, we used a strong loss-of-function allele of *lid* (*lid^10424^*) (Gildea et al., 2000; Li et al., 2010). The *lid^10424^/Df* hemizygous flies (referred to hereafter as *lid*) are pupal lethal with rare adult escapers. We analyzed H3K4me3 levels using immunoblot on testes isolated from third instar larvae of *lid* males and compared to wild-type (wt) third instar larval testes. Using antibodies against H3K4me3 and H3 as a control we found that loss of *lid* leads to an approximately 4-fold increase in H3K4me3 levels (Fig. S1A-B).

To explore the expression pattern of Lid in testis, we performed immunostaining using anti-Lid antibody (Secombe et al., 2007) in wt testes. The Lid protein was detected in the nuclei of cells throughout the testis with strong signals in the germline (Fig. S1C-C″). To determine the role of *lid* in the male GSC niche, we analyzed testes isolated from third instar larvae of *lid* males. We detected a marked decrease of GSC number in *lid* testes compared to wt testes. The decrease in GSC number was visible in immunostained images when comparing wt testes (Fig. 1B) with *lid* testes (Fig. 1C, dots). When quantified, we found that wt testes had an average of 9.7±1.6 GSCs compared to an average of 6.03±1.4 GSCs in *lid* testes (*P*<0.01; Fig. 1D). Furthermore, GSCs appeared to be detached from the hub cells in the majority of *lid* testes (Fig. 1C, arrow). These results suggest that Lid is required to maintain GSCs at the *Drosophila* testis niche.

Due to adult lethality we next analyzed the *lid* mutant phenotype in adult testes using the FLP-mediated FRT recombination system (Xu and Rubin, 1993). We generated mutant clones for *lid* using the strong loss-of-function allele *lid^k6801^* (Gildea et al., 2000; Secombe et al., 2007). The percentage of testes with at least one *lid* GSC clone declined over time compared to the wt control (Fig. 1E), suggesting that Lid is required in the germline to maintain GSCs at the *Drosophila* testis niche.

To confirm this conclusion, we used different cell-type specific Gal4 drivers in combination with a *UAS-lid* small hairpin microRNA (shmiRNA) (Ni et al., 2011) to knockdown *lid* in a cell type-specific manner. Knockdown of *lid* exclusively in early stage germ cells using *nanos* (*nos*)*-Gal4* (Van Doren et al., 1998) led to a significant decrease in GSC number (Fig. 1F-G, dots; 1H). By contrast, knockdown of *lid* using the early cyst cell driver *c587-Gal4* (Manseau et al., 1997) (Fig. 1H and Fig. S2A-B) or the hub driver *upd-Gal4* (Boyle et al., 2007) (Fig. 1H and Fig. S2C-D) did not lead to a change in GSC number. These results demonstrate that normal function of Lid is required in early stage germ cells including GSCs, but not in somatic gonadal cells, to prevent GSC loss at the testis niche.

In addition to its role in GSC maintenance, we also found that Lid is required for proper hub architecture (compare hub region in Fig. 1B and C). Loss of *lid* led to a dramatic increase in hub size compared to wt testes (Fig. S3A). The increase in hub size is a secondary effect due to GSC loss, as reported previously (DiNardo et al., 2011; Gonczy and DiNardo, 1996; Monk et al., 2010; Tazuke et al., 2002). Consistently, this phenotype was recapitulated by knocking down *lid* in germ cells but not in somatic gonadal cells (Fig. S3B).

### Lid is required to maintain GSC proliferation and prevent premature differentiation

**Fig. 2.**
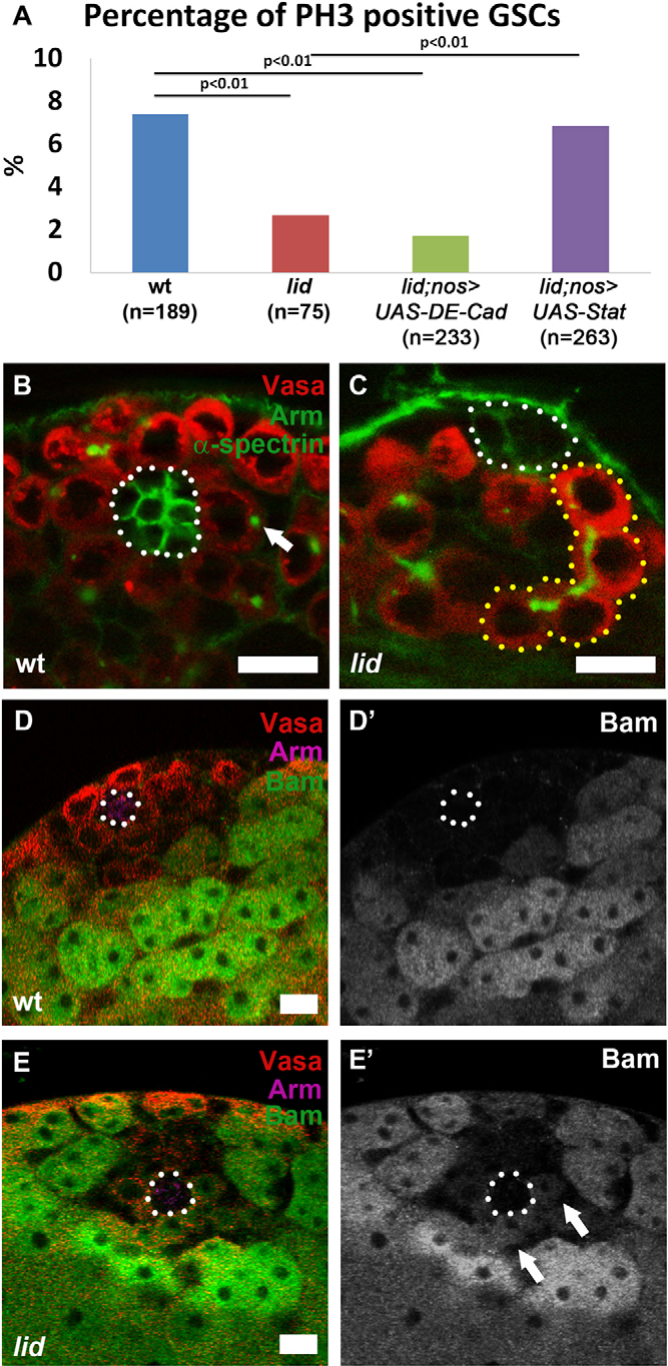
**Lid is required to maintain GSC proliferation and prevent premature differentiation.** (A) Percentage of PH3 positive GSCs in testes from males of the following genotypes: wt (7.4%); *lid* (2.7%); *lid, UAS-DE-Cadherin; nos-Gal4* (1.7%); and *lid, UAS-Stat92E; nos-Gal4* (6.8%). *P-*value calculated using Fisher's test. (B,C) Immunostaining using antibodies against Vasa (red), Arm (green), and α-spectrin (green) in wt (B) and *lid* (C) testes. Arrow points to round spectrosome in (B). 4-cell spermatogonia cyst at the hub is outlined (yellow dotted line) in (C). Hub area is outlined (white dotted line). (D-E′) Immunostaining with antibodies against Vasa (red), Arm (magenta), and Bam-GFP (green) in *Bam-GFP* (D,D′) and *lid; Bam-GFP* (E,E′) testes. Arrows point to Bam-expressing GSCs in (E′). Hub area is outlined (white dotted line). Scale bars: 10 µm.

To determine the mechanism leading to GSC loss in the *lid* testes, we used phospho-Histone H3 (PH3) immunostaining to assess the mitotic index of GSCs. We observed a significant decrease in the mitotic activity of GSCs in *lid* testes compared to wt control (Fig. 2A, first two columns), suggesting a role for Lid in maintaining GSC proliferation. While the GSC mitotic index in wt third instar larvae is comparable to what was reported previously (Parrott et al., 2012), it is worth noting that it is higher than what was reported for GSCs from wt adult flies (Sheng and Matunis, 2011; Yadlapalli et al., 2011; Yadlapalli and Yamashita, 2013). This is likely because GSCs undergo an expansion at the third instar larval developmental stage. Indeed when we counted H3T3P-positive GSCs in adult testes from *nos-Gal4* control, we detected a lower mitotic index at 2.5% (15/607 total GSCs). In comparison, GSCs in *nos>lid shmiRNA* testes showed an approximate ∼2-fold reduction at 1.4% (8/579 total GSCs). We further confirmed this reduction by using another mitosis-enriched H3S10P antibody, which revealed ∼3-fold reduction of H3S10P-positive GSC ratio in *nos>lid shmiRNA* testes (3.2%, 9/283 total GSCs) compared to the *nos-Gal4* control (9.3%, 30/324 total GSCs).

We next asked whether the loss of GSCs is accompanied by premature differentiation. Fusomes are branched cytoplasmic structures, comprised of membrane skeletal proteins such as α- and β-spectrin, that connect germ cells in a cyst (Deng and Lin, 1997). When we used antibody against α-spectrin, the fusome appears spherical (called spectrosome) in all GSCs (Fig. 2B, arrow) and becomes branched in further differentiated spermatogonia of wt testes (*n*=44). By contrast, we observed spermatogonial cysts containing up to four interconnected germ cells in direct association with the hub region (Fig. 2C, yellow dotted outline) in 33% of the *lid* testes (*n*=52). This phenomenon could be due to premature differentiation of GSCs or dedifferentiation of spermatogonial cells. It has been reported that dedifferentiated spermatogonial cells tend to have a higher percentage of misoriented centrosomes when homing back to the niche (Cheng et al., 2008). We labeled centrosomes using antibody against γ-tubulin, a major component of the centrosome (Yamashita et al., 2003) and found that the percentage of GSCs with misoriented centrosomes was not significantly different in *lid* (6.3%, *n*=59) compared to wt testes (7.1%, *n*=104) (*P*>0.05). In addition to misoriented centrosomes, it has been reported that transiently disintegrating fusome remnants are detectable in dedifferentiated spermatogonial cells (Brawley and Matunis, 2004; Cheng et al., 2008). Using antibody against α-Spectrin, we did not observe any disintegrating fusome remnants in GSCs from *lid* testes (*n*=52). Taken together, these data suggest that GSCs in *lid* testes are lost due to decreased proliferation and premature differentiation.

Since 33% of *lid* testes contain spermatogonial cysts with branched fusomes next to the hub, the counting of GSCs as Vasa-positive cells that are directly associated with the hub region in *lid* testes might be an overestimation. Therefore we re-quantified the number of GSCs in *lid* testes: we defined GSCs as Vasa-positive, spectrosome-containing cells in direct association with the hub cells. Indeed, we find that using this method, the average number of GSCs is 5.86±1.69 per *lid* testis (*n*=52), which is slightly, but not significantly, lower than the 6.03±1.4 GSCs reported in Fig. 1D. These results suggest that the germ cells that are part of differentiating spermatogonial cysts do not contribute significantly to the overall GSC scoring in *lid* testes.

To further understand the GSC premature differentiation phenotype, we performed immunostaining using a germ cell differentiation marker, Bag of marbles (Bam) (Gonczy et al., 1997), in both wt and *lid* testes. Bam is normally expressed in four-to-16-cell spermatogonial cells (Gonczy et al., 1997). Consistently, using a Bam-GFP reporter transgene (Chen and McKearin, 2003), we found that the GFP signal is only detectable in differentiating spermatogonial cysts away from the hub in wt testes (*n*=20; Fig. 2D-D′). By contrast, Bam-GFP-positive cells were detected in direct association with the hub cells in 15% of the *lid* testes (*n*=20; Fig. 2E-E′, arrows in 2E′), further indicating that Lid is required to prevent GSCs from undergoing premature differentiation.

### Lid acts in germ cells to maintain the proper level of the Stat92E transcription factor

**Fig. 3.**
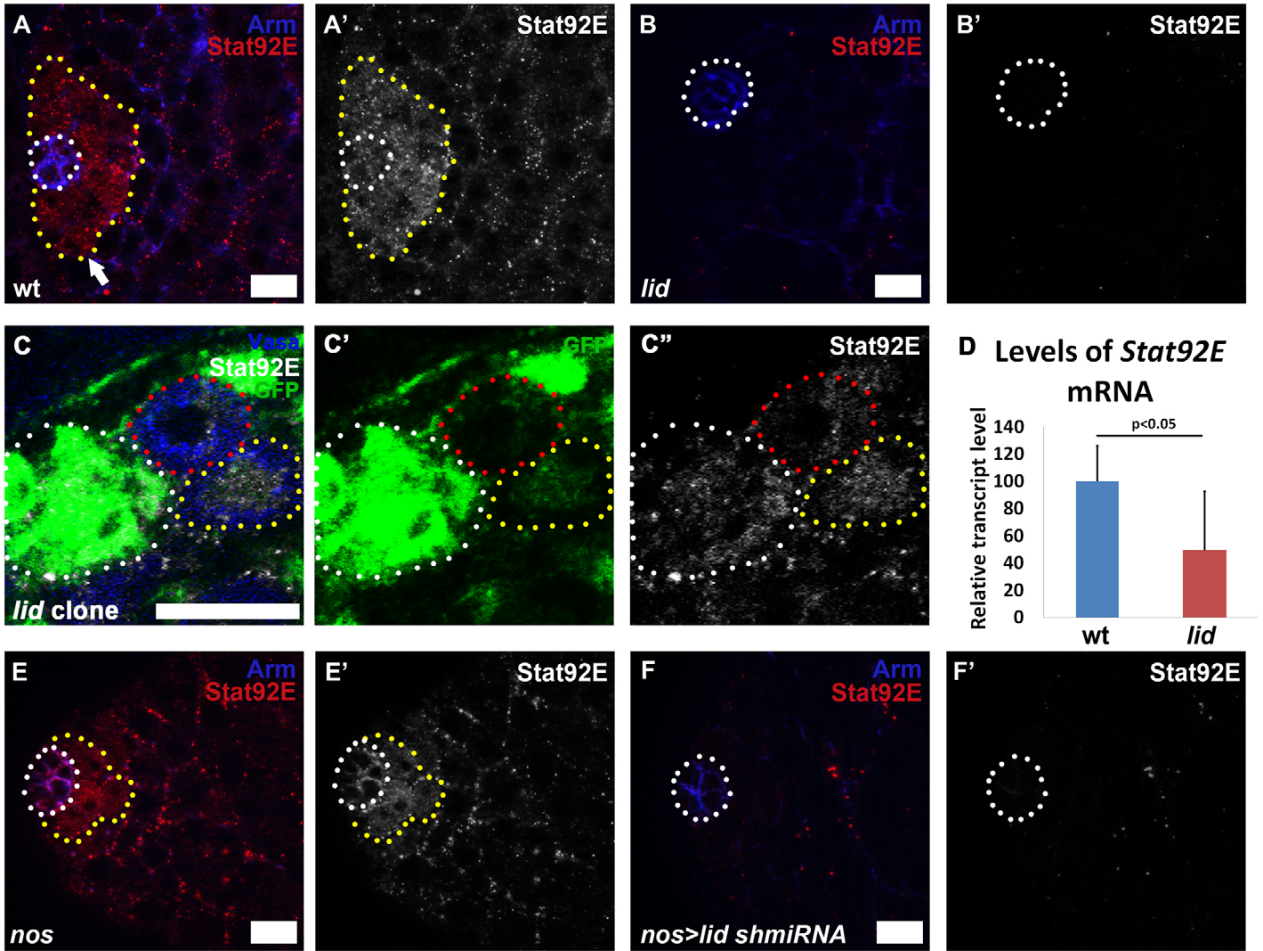
**Lid acts in germ cells to maintain the proper level of the Stat92E transcription factor.** (A-B′,D-E′) Immunostaining with antibodies against Arm (blue) and Stat92E (red) in wt (A,A′), *lid* (B,B′), *nos-Gal4* (E,E′), and *nos-Gal4/UAS-lid shmiRNA* (F,F′) testes. Stat92E-expressing cells are outlined (yellow dotted line) in (A,A′,E,E′). Arrow points to a Stat92E-expressing gonialblast in (A). Hub area is outlined (white dotted line). (C-C″) Immunostaining with antibodies against Vasa (blue), GFP (green) and Stat92E (white) in testis with *lid* GSC clones. Hub area is outlined (white dotted line), a GFP-negative *lid* GSC clone is outlined (red dotted line), a GFP-positive control GSC is outlined (yellow dotted line). (D) *Stat92E* mRNA levels measured by qRT-PCR in three independent biological replicates, normalized by *RpL32*. *P-*value calculated using Student's *t*-test. Error bars represent s.d. The Stat92E antibody may have a high detection threshold as it shows complete loss of Stat92E immunostaining signal in *lid* mutant testes and *lid* mutant GSC clones. Scale bars: 10 µm.

The JAK-STAT pathway is one of the major pathways that maintain stem cell activity and identity in the testis niche. Testes depleted of *Stat92E* display severe loss of both CySC and GSC populations (Kiger et al., 2001; Leatherman and DiNardo, 2008, 2010; Tulina and Matunis, 2001). In wt testes, Stat92E is highly enriched in GSCs and some of their immediate daughter cells, but rapidly declines in further differentiated cells (Fig. 3A-A′). By contrast, *lid* testes had no detectable Stat92E signal (Fig. 3B-B′), even though testes from both genotypes were immunostained together, mounted on the same slide and imaged using the same microscope settings.

To confirm the decrease of Stat92E in *lid* GSCs, we stained testes containing *lid* GSC clones with antibody against Stat92E. We observed lower Stat92E immunostaining signal in *lid* GSCs compared to the neighboring control GSCs (Fig. 3C-C″). In addition, we used quantitative reverse transcription PCR (qRT-PCR) to measure the *Stat92E* mRNA levels. The *Stat92E* transcript level in *lid* testes decreased to ∼50% of the level in the wt control when a constitutively expressed *RpL32* gene was used as an internal control (Fig. 3D). It is worth noting that halving the level of Stat92E in *Stat92E/+* testes did not cause any obvious phenotypes. Therefore it is likely that the decrease of *Stat92E* transcript is higher than 50% in *lid* GSCs, but using the whole tissue with mixed cell types and different stages of germ cells to measure *Stat92E* mRNA levels compromised this effect.

Finally, we compared *nos*>*lid shmiRNA* testes with the *nos-Gal4* control using the similar experimental strategy as described above. We also found a marked decrease of the Stat92E immunostaining signal at the niche in the *nos*>*lid shmiRNA* testes (Fig. 3F-F′) compared to the *nos-Gal4* control (Fig. 3E-E′). In summary, these results demonstrate that Lid acts in germ cells to maintain proper expression of the Stat92E transcription factor. Interestingly, the decrease of Stat92E in *nos*>*lid shmiRNA* testes is also accompanied by a decrease of Stat92E signals in the hub cells (Fig. 3F′ vs 3E′), suggesting a potential non-cell autonomous role for Lid in maintaining JAK-STAT signaling in the hub cells.

### Lid regulates Stat92E in GSCs to maintain proliferation and prevent premature differentiation

**Fig. 4.**
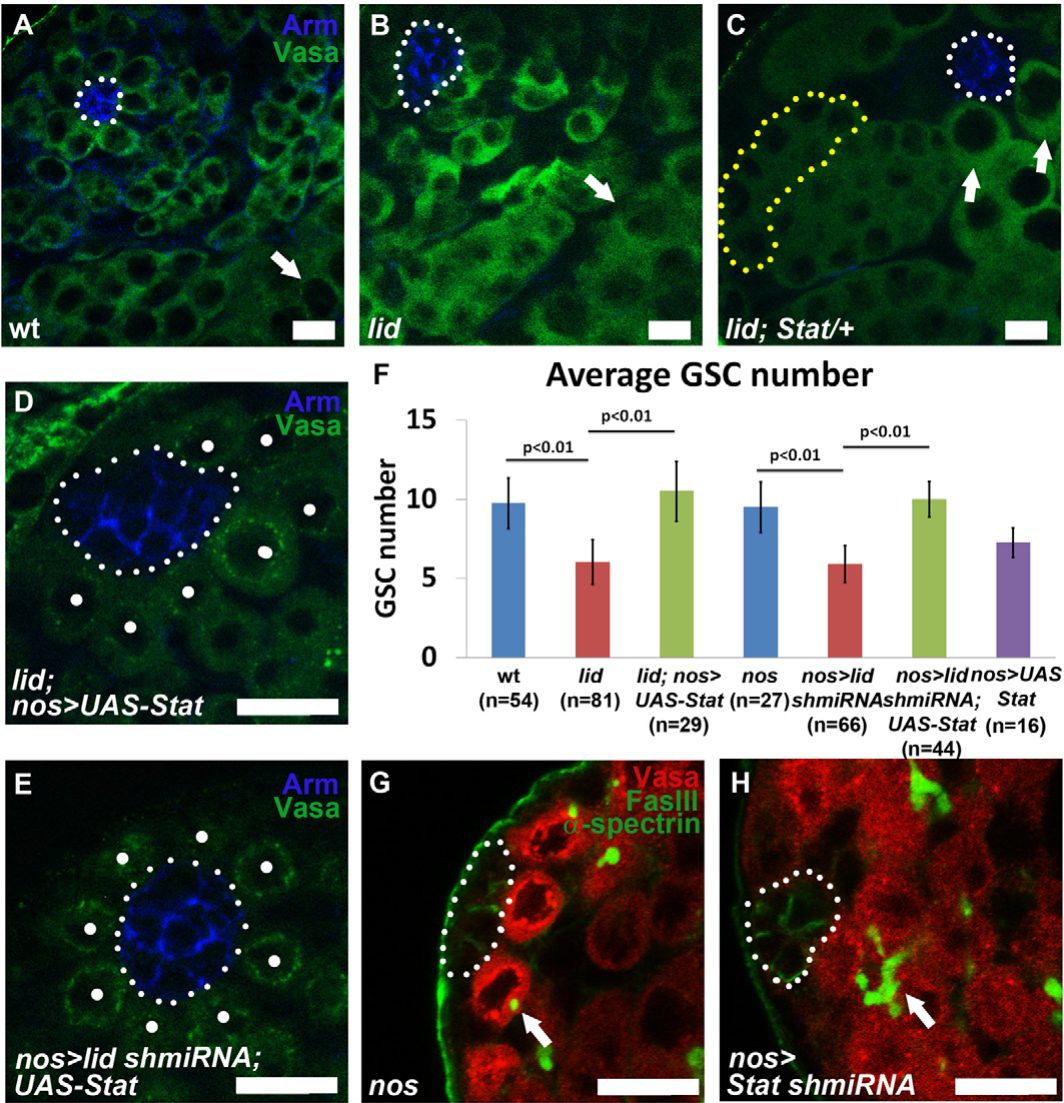
**Lid regulates Stat92E in GSCs to maintain their proliferation and prevent premature differentiation.** (A-E) Immunostaining using antibodies against Arm (blue) and Vasa (green) in wt (A), *lid* (B), and *lid; Stat92E/+* testes (C). Arrows point to spermatocytes. 8-cell spermatogonia cyst is outlined (yellow dotted line) in (C). (D) *lid, UAS-Stat92E; nos-Gal4* with rescued GSC number. (E) *nos-Gal4/UAS-lid shmiRNA; UAS-Stat92E* testis with rescued GSC number. Dots indicate GSCs which we identified as Vasa-labeled cells in direct contact with the hub. Hub area is outlined (white dotted line). (F) Quantification of average GSC number in testes from males of the following genotypes: wt (9.7±1.6); *lid* (6.03±1.4); *lid, UAS-Stat92E; nos-Gal4* (10.5±1.9); *nos-Gal4* control (9.5±1.6); *nos-Gal4/UAS-lid shmiRNA* (5.9±1.1); *nos-Gal4/UAS-lid shmiRNA; UAS-Stat92E* (10±1.1) and *nos-Gal4; UAS-Stat92E* (7.3±0.9). *P-*values calculated using Student's *t*-test. Error bars represent s.d. (G,H) Immunostaining with antibodies against Vasa (red), FasIII (green), and α-spectrin (green) in *nos-Gal4* (G) and *nos-Gal4; UAS-Stat shmiRNA* (H) testes. Arrows point to round spectrosome in (G) and branched fusome in (H). Scale bars: 10 µm.

Because Lid is required for proper expression of Stat92E, we tested whether further reducing Stat92E levels could enhance *lid* mutant phenotype. By removing one copy of *Stat92E* using a strong loss-of-function allele (*Stat92E^06346^*) (Hou et al., 1996) at *lid* mutant background, we found a significant enhancement of the GSC loss and premature differentiation phenotypes. In wt testes, after GSC asymmetric cell division, the differentiating daughter cell called gonialblast (GB) undergoes four rounds of mitosis and then enters meiosis. During the elongated G2 phase of meiosis, male germ cells grow 25 times in volume as spermatocytes. Spermatocytes are distinguishable from spermatogonia based on their large size and distinct nuclear morphology (White-Cooper et al., 1998). In wt testes, spermatocytes are distant from the niche and never observed in close proximity with the hub cells (Fig. 4A, arrow). In *lid* mutant testes, although we found differentiated spermatogonial cysts in direct contact with hub cells, we have never observed spermatocytes in direct contact with the hub cells (Fig. 4B, arrow). However, in drastic contrast, we found that spermatocytes are in direct association with the hub cells in 100% of *lid; Stat92E/+* testes (Fig. 4C, arrows). In these testes, the earliest stage of germ cells we could detect is the eight-cell spermatogonia (Fig. 4C, yellow dotted outline). These results demonstrate that both the severity and the penetrance of the *lid* mutant phenotype are enhanced by loss of one copy of *Stat92E*, suggesting that Lid acts in synergy with Stat92E to prevent GSC premature differentiation. On the other hand, the enhanceable phenotype of *lid* mutant is consistent with the measurement of *Stat92E* transcript level in *lid* mutant testes (Fig. 3D), suggesting a partial loss of *Stat92E* in *lid* mutant testes. These results also explain the reason why GSCs mutant for Stat92E using a strong loss-of-function allele (*Stat92E^06346^*) are lost at a more rapid rate (Tulina and Matunis, 2001) compared to *lid* mutant GSCs (Fig. 1E).

To explore whether Stat92E acts downstream of Lid, we next examined whether overexpression of a *Stat92E* cDNA is sufficient to rescue the *lid* mutant defects. We found that driving a *UAS-Stat92E* transgene (Bach et al., 2003) using *nos-Gal4* rescued both GSC loss (Fig. 4D,F 3rd vs 2nd column) and premature differentiation phenotype of *lid* mutant testes: the percentage of testes with spermatogonial cysts next to the hub decreased from 33% (*n*=52) in *lid* mutant to 5% (*n*=40) in *lid; nos>UAS-Stat* testes. In addition to *lid* mutant testes, we tested whether *nos>UAS-Stat* can rescue *nos>lid shmiRNA* phenotype in which the function of Lid is only compromised in germ cells. We found that *nos>UAS-Stat* rescued GSC loss in *lid* knockdown testes (Fig. 4E,F, 6th vs 5th column). As a control for both experiments, we drove *UAS-Stat92E* expression using the same *nos-Gal4* at a wt background and did not observe an increase in GSC number. This result is consistent with the previous report that increasing Stat92E level in the germline using an activated allele of *jak* (*Hop^Tuml^*) does not lead to any ectopic phenotype (Fig. 4F, last column) (Leatherman and DiNardo, 2008). In summary, these results demonstrate that Stat92E acts downstream of Lid in maintaining GSC proliferation and preventing their premature differentiation.

Based on our data on Lid's function, we hypothesize that Lid is required for normal expression of Stat92E, which is required for GSC maintenance. Then loss of *Stat92E* in the germline should phenocopy the *lid* mutant phenotype. To test this hypothesis more directly, we compromised Stat92E expression by driving a *UAS-Stat92E shmiRNA* exclusively in early germ cells using the same *nos-Gal4* driver. A previous study reported that knockdown of germline *Stat92E* using RNA interference (RNAi) resulted in testes with displaced GSCs that clustered next to CySCs instead of associating with the hub (Leatherman and DiNardo, 2010). While the detachment of GSCs was not observed in the *nos-Gal4* control (Fig. S4A-A′), we did observe a similar phenotype in 30% of *nos>UAS-Stat92E shmiRNA* testes (*n*=33; Fig. S4B-B′). In addition, we found that knockdown of *Stat92E* specifically in the germline led to phenotypes similar to *lid* mutant testes in which differentiated spermatogonial cysts directly contact the hub cells in 48% of testes (*n*=33; Fig. 4H, arrow). By contrast, only spectrosome-containing GSCs were located next to the hub in all *nos-Gal4* control testes (*n*=30; Fig. 4G, arrow). The premature differentiation phenotype is more penetrant in *UAS-Stat92E shmiRNA* testes (48%) compared to that in *lid* mutant testes (33%), consistent with the partial loss of *Stat92E* phenotype in *lid* mutant testes as discussed previously. Taken together, these results suggest that both Lid and Stat92E act in the germline to prevent GSCs from undergoing premature differentiation.

### Ptp61F and DE-Cadherin interact with Lid to maintain GSC number

**Fig. 5.**
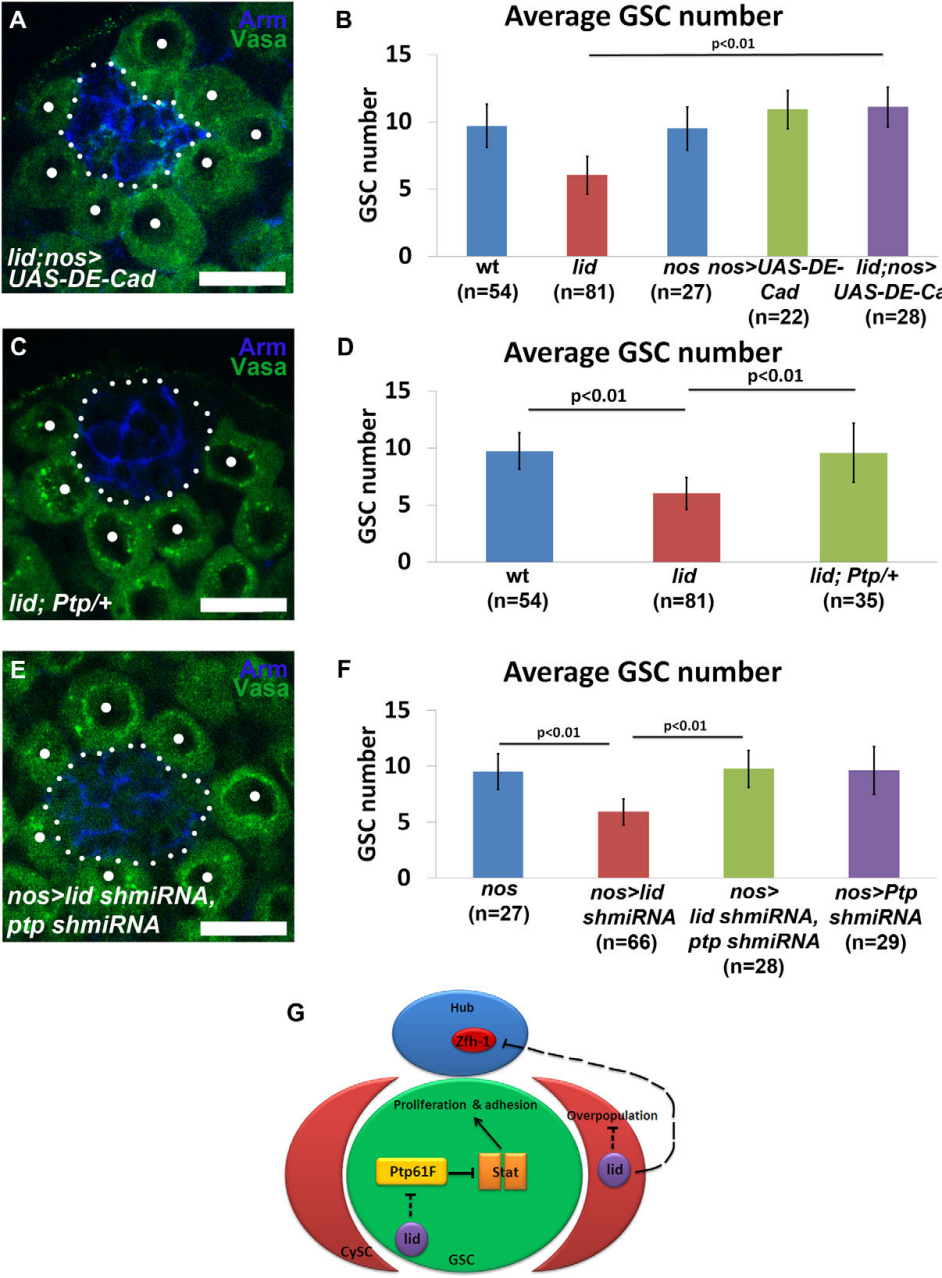
**Ptp61F and DE-Cadherin interact with Lid to maintain GSC number and model of Lid function in the testes niche.** (A,C,E) Immunostaining using antibodies against Arm (blue) and Vasa (green) in *lid, UAS-DE-Cadherin; nos-Gal4* (A); *lid; Ptp61F/+* (C) and *nos-Gal4/UAS-lid shmiRNA; UAS-Ptp61F shmiRNA* (E) testes. Dots indicate GSCs which we identified as Vasa-labeled cells in direct contact with the hub. Hub area is outlined (white dotted line). Scale bars: 10 µm. (B) Quantification of average GSC number in testes from males of the following genotypes: wt (9.7±1.6); *lid* (6.03±1.4); *nos-Gal4* control (9.5±1.6); *UAS-DE-Cadherin; nos-Gal4* (10.9±1.4); and *lid, UAS-DE-Cadherin; nos-Gal4* (11.1±1.4). (D) Quantification of average GSC number in testes from males of the following genotypes: wt (9.7±1.6); *lid* (6.03±1.4); *lid; Ptp61F/+* (9.3±2.4). (F) Quantification of average GSC number in testes from males of the following genotypes: *nos-Gal4* control (9.5±1.6), *nos-Gal4/UAS-lid shmiRNA* (5.9±1.1), *nos-Gal4/UAS-lid shmiRNA; UAS-Ptp61F shmiRNA* (9.7±1.6), and *nos-Gal4; UAS-Ptp61F shmiRNA* (9.6±2.1). (G) Outline of Lid function in the testis niche. Lid cell-autonomously regulates the JAK-STAT signaling pathway in germ cells to maintain GSC proliferation. Lid is also required in CySCs to maintain niche architecture. *P-*values calculated using Student's *t*-test. Error bars represent s.d.

A previous study reported that Stat92E regulates adhesion of GSCs to the hub by maintaining DE-Cadherin levels (Leatherman and DiNardo, 2010). The increased detachment of GSCs in *lid* testes (Fig. 1C, arrow) could therefore be due to decreased DE-Cadherin because of compromised Stat92E (Fig. 4). Indeed, we find that driving wild-type DE-Cadherin (*UAS-DE-Cad^DEFL^*) (Inaba et al., 2010) using *nos-Gal4* rescued GSC loss in *lid* testes (Fig. 5A-B). However, the rescue of GSC number was not accompanied by restoring GSC mitotic activity measured by anti-PH3 immunostaining (Fig. 2A, 3rd column). While Lid could control DE-Cadherin levels directly or indirectly by regulating Stat92E levels, our data suggest that *lid* mutant GSCs have more defects than decreased DE-Cadherin levels. By contrast, both the number and the mitotic index of GSCs in *lid; nos>UAS-Stat* testes were restored to the level in wt testes (Fig. 2A, 4th column). Therefore our results demonstrate that Lid acts in synergy with Stat92E to maintain GSCs by regulating their proper proliferation.

Since Lid is a demethylase that erases the H3K4me3 active histone modification, it is likely that Lid acts as a transcriptional repressor (Secombe and Eisenman, 2007). Then a JAK-STAT pathway negative regulator could be repressed by Lid which would lead to decreased Stat92E level when Lid function is compromised. To test this, we investigated genetic interactions between Lid and protein tyrosine phosphatase (Ptp61F), a negative regulator of the JAK-STAT pathway (Baeg et al., 2005). Ptp61F is a tyrosine phosphatase that targets Stat92E and potentially Hop to inhibit their activity (Baeg et al., 2005). We found that removing one copy of *Ptp61F* using a loss-of function allele (*Ptp61F^PBac^*) was sufficient to suppress the GSC loss phenotype in *lid* testes (Fig. 5C-D). Furthermore, when we drove *UAS-Ptp61F shmiRNA* using the *nos-Gal4* driver, we observed suppression of the GSC loss phenotype in *nos>lid shmiRNA* testes (Fig. 5E,F, 2nd and 3rd columns). As a control, the *UAS-Ptp61F shmiRNA* driven by the *nos-Gal4* did not lead to any significant change of GSC number (Fig. 5F, last column), consistent with the previous observation that overexpression of Stat92E in germline does not lead to any ectopic phenotype (Fig. 4F, last column). Therefore in both *lid* mutant and germ cell-specific *lid* knockdown testes, Ptp61F acts antagonistically with Lid in maintaining GSCs.

## DISCUSSION

In this study, we identify a new epigenetic regulator of the JAK-STAT signaling pathway in the *Drosophila* testis niche (Fig. 5G). Lid acts in the germ cells to regulate Stat92E levels, maintain GSC proliferation and prevent premature differentiation of GSCs. Our results demonstrate an essential role for a histone demethylase in regulating a major signaling pathway to maintain stem cell activity *in vivo*.

### Lid is a new epigenetic regulator of the JAK-STAT signaling pathway

Mechanisms responsible for the maintenance of stem cell activities are vital as disruption in these mechanisms is implicated in many types of disease including cancer. The JAK-STAT pathway is one of these mechanisms that play a critical role in stem cell maintenance across a range of species. Abnormalities in this pathway have been reported to lead to tumorigenesis in mammals (Bowman et al., 2000; Leeman et al., 2006). Here we report that Lid maintains GSCs at the *Drosophila* testis niche by regulating JAK-STAT pathway activity. Loss of *lid* leads to decreased Stat92E levels and premature GSC differentiation. Our data provide a novel epigenetic mechanism that regulates the JAK-STAT signaling pathway in an endogenous stem cell niche. It will be imperative to determine whether these links between epigenetic and transcriptional regulation occur in other stem cell systems.

### The JAK-STAT pathway is required cell autonomously for GSC maintenance

The JAK-STAT pathway in the *Drosophila* testis niche is a major pathway required for both GSCs and CySCs activities. Several lines of evidence in our study support a direct role of Stat92E in maintaining GSC identity and activity. First, germline-specific knockdown of *Stat92E* leads to premature differentiation of GSCs. Second, expressing a Stat92E cDNA in germ cells rescues GSC loss, decreased mitotic activity, and premature differentiation phenotypes in *lid* testes. Finally, expression of *DE-Cadherin* in *lid* GSCs was insufficient to restore GSC mitotic index. Therefore we hypothesize that STAT activity is required in GSCs to maintain GSC number and proliferation and prevent premature differentiation.

### Distinct biological functions of histone demethylases

In this study, we report an important cell autonomous function of H3K4me3-specific histone demethylase Lid in male GSC maintenance in *Drosophila*, which is different from the H3K27me3-specific histone demethylase dUTX reported to have a non-cell autonomous role in the germline (Tarayrah et al., 2013). While both demethylases are required to regulate stem cell activities, Lid mainly regulates GSC while dUTX regulates CySC activities at the niche. Interestingly, both Lid and dUTX maintain the activities of their target stem cell population by regulating the JAK-STAT pathway. However, while dUTX controls the transcription of Socs36E, an inhibitor of JAK-STAT in CySCs, Lid targets another JAK-STAT inhibitor Ptp61F in the germline.

Interestingly, we did observe a comparable role for Lid in CySCs. Using an antibody against zinc finger homeodomain 1 (Zfh1) to label CySCs and early cyst cells, we detected niche architectural defects in *lid* testes. In wt testes, Zfh1-expressing CySCs surround GSCs and extend thin protrusions toward the hub forming a clear rosette structure (Fig. S5A-A′, arrows). However, in *lid* testes, Zfh1-expressing cells had nuclei that directly contacted the hub surface (Fig. S5B-B′, white arrows). Furthermore, knockdown of *lid* using the cyst cell driver *c587-Gal4*, but not the germ cell driver *nos-Gal4* or the hub cell driver *upd-Gal4*, led to the overpopulation of Zfh1-expressing cells around the hub (Fig. S5C-D, compare white arrows). This suggests that Lid is required in CySCs and/or early cyst cells to prevent overpopulation of Zfh1-expressing cells around the hub.

Our results also revealed that 61% (*n*=76) of *lid* testes had hub cells that ectopically expressed Zfh1 compared to only 14% (*n*=41) of wt testes exhibiting this phenotype (Fig. S5B-B′, yellow arrows). Interestingly, knockdown of *lid* using the cyst cell driver *c587-Gal4*, but not the germ cell driver *nos-Gal4* or the hub cell driver *upd-Gal4*, led to ectopic Zfh1 expression in the hub (Fig. S5D, yellow arrow; Fig. S5E). These data indicate that the function of Lid in CySCs or early cyst cells is essential to prevent Zfh1 from ectopically turning on in hub cells. These results reveal dynamic communication among the different cell types in the testis niche where CySCs send feedback to hub cells to maintain proper gene expression. We do not however understand the biological relevance of ectopic Zfh1 expression in hub cells. When we drive the *UAS-Zfh1* transgene using the *upd-Gal4* driver we do not observe any apparent phenotypes in niche architecture and function, therefore the biological consequence of ectopic Zfh1 expression remains unclear.

In summary, our results emphasize the importance of studying chromatin regulators' functions *in vivo* in the context of cell-cell communication, because their activities connect cell intrinsic mechanisms such as transcription with extrinsic signaling.

## MATERIALS AND METHODS

### Fly stocks

Flies were raised on standard yeast/molasses medium at 25°C. The following stocks were used: *lid^10424^* (Bloomington Stock Center, BL-12367), *lid^k06801^*(Bloomington Stock Center, BL-10403), *w^1118^; Df(2L) BSC184* (Bloomington Stock Center, BL-9612), *lid^k06801^,FRT40A* (Bloomington Stock Center, BL-111088), *UAS-lid shmiRNA* (Valium 10, TRiP.HM05155 from Bloomington Stock Center, BL-28944), *UAS-lid shmiRNA* (Valium 22, TRiP.GL00612 from Bloomington Stock Center, BL-36652), *upd-Gal4* (from D. Harrison, University of Kentucky, Lexington, KY, USA), *nanos-Gal4* (from M. Van Doren, Johns Hopkins University, Baltimore, MD, USA), *c587- Gal4* (from A. Spradling, Carnegie Institution Department of Embryology, Baltimore, MD, USA), *y,w; Ubi-GFP, Ubi-GFP, FRT40A* (Bloomington Stock Center, BL-5189), *hs-FLP^122^* (Bloomington Stock Center, BL- 33216), *w;* *FRT40A* (Bloomington Stock Center, BL-5756), *w;;* *Bam-GFP* (from D. McKearin, University of Texas Southwestern Medical Center, Dallas, TX, USA), *Stat92E^06346^* (from N. Perrimon, Harvard Medical School, Boston, MA, USA), *UAS-Stat92E* (from E. Bach, New York University School of Medicine, New York, NY, USA), *UAS-Stat shmiRNA* (Valium 20, TRiP.HMS00035 from Bloomington Stock Center, BL-3367), *UAS-DE-Cad^DEFL^* (from Y. Yamashita, University of Michigan, Ann Arbor, MI, USA), *UAS- Ptp61F shmiRNA* (Valium 20, TRiP.HMS00421 from Bloomington Stock Center, BL-32426), *Ptp61F^PBac^* (Bloomington Stock Center, BL-17698), and *Act5C<stop<Gal4, UAS-GFP* (from J. Secombe, Albert Einstein College of Medicine, Bronx, NY, USA).

### Clonal induction

*lid^k06801^* clones were generated using the FLP/FRT recombination system. The flies used had the following genotypes: *hs-FLP^122^; Ubi-GFP, Ubi-GFP, FRT40A/lid^k06801^ FRT40A* or *hs-FLP^122^; Ubi-GFP, Ubi-GFP*, *FRT40A/FRT40A*. The clones were induced by heat shocking pupae on days eight and nine for two hours at 37°C. After the second heat shock, flies were placed at 25°C and dissected and stained one, three and seven days after clone induction.

### Immunofluorescence staining

Testes were dissected in 1× PBS and fixed in 4% formaldehyde for 30 min. For immunostaining, testes were incubated with primary antibodies overnight at 4°C, followed by washes in 1× PBST and incubation with secondary antibodies for two hours at RT. The following primary antibodies were used: rabbit anti-Lid (1:1000; from Julie Secombe, Albert Einstein College of Medicine, Bronx, NY, USA); mouse anti-Armadillo [1:100; developed by Eric Wieschaus, Princeton University, Princeton, NJ, USA, and obtained from Developmental Studies Hybridoma Bank (DSHB)]; rat anti-Vasa (1:100; developed by Allan Spradling and Dianne Williams and obtained from DSHB); rabbit anti-Vasa (1:100; Santa Cruz, sc-30210); chicken anti-GFP (1:1000; Abcam, #13970); rabbit anti-Stat92E (1:800; from Denise Montell, Johns Hopkins School of Medicine, Baltimore, MD, USA); rabbit anti-phospho-Histone H3 (Thr3) (1:200; Millipore, #05-746R); mouse anti-α-spectrin (1:50; obtained from DSHB); mouse anti-γ-tubulin (1:100, Sigma, GTU-88); mouse anti-FasIII (1:50; obtained from DSHB, 7G10); rabbit H3K4me3 (1:200; Cell Signaling, #9751S); rabbit anti-Zfh1 (1:5000; from Ruth Lehmann, Skirball Institute of Biomolecular Medicine, NY, USA). Alexa Fluor 488, 568 and 633-conjugated Goat anti-mouse, anti-rabbit, and anti-rat secondary antibodies were used (1:200; Molecular Probes/Invitrogen).

### Isolation of total RNA and quantitative reverse transcription polymerase chain reaction (qRT–PCR)

Total RNA was collected and isolated from wild-type (wt) and *lid* third instar larval testes using TRIzol reagent (Invitrogen, #15596-018) according to the manufacturer's instructions. The yield and quality of the RNA was determined using a NanoDrop spectrometer (NanoDrop Technology, San Diego, CA, USA). Reverse transcription was performed using the RevertAid First Strand cDNA Synthesis Kit (Fermentas, #K1621). RNA Transcript levels were then measured using SYBR Green PCR Master Mix (Fermentas, #K0221) and normalized by *RpL32*. Sequences of primers used for qRT-PCR were as follows:

AAGGTGAGTGATTTGCTGTGCTGC (*Stat92E-*forward)

CAACAAGCGAGCATGAGAATGCCA (*Stat92E-*reverse)

CATGCTGCCCACCGGATTCAAGAAG (*RpL32-*forward)

CTCGTTCTCTTGAGAACGCAGGCGA (*RpL32-*reverse)

### Western blotting

Testes were dissected in 1× PBS. Samples were homogenized in 25 μl 1× PBS containing 4× DualColor Protein Loading Buffer (Fermentas, #R1011), boiled and loaded onto a 4-20% gradient SDS-PAGE gel (Novex, EC6065). Primary antibodies anti-H3K4me3 (1:1000; Cell Signaling, #9751S) and anti-H3 (1:5000; Abcam, ab1791) were used.

### Statistical analysis

Statistical significance was calculated using two-tailed Student's *t*-test or Fisher's test. *P*-values are indicated in figures or in figure legends. Error bars indicate standard deviation (s.d.).
